# Robot-assisted thoracoscopic surgery vs. sternotomy for thymectomy: A systematic review and meta-analysis

**DOI:** 10.3389/fsurg.2022.1048547

**Published:** 2023-01-06

**Authors:** Cheng-qian Wang, Jie Wang, Fei-yu Liu, Wei Wang

**Affiliations:** ^1^ Department of Thoracic Surgery, The Affiliated Yantai Yuhuangding Hospital of Qingdao University, Yantai, China; ^2^ The Second Medical College, Binzhou Medical University, Yantai, China; ^3^ Department of Pharmacy, The Affiliated Yantai Yuhuangding Hospital of Qingdao University, Yantai, China

**Keywords:** robot-assisted thoracoscopic surgery, sternotomy, thymectomy, systematic review, meta-analysis

## Abstract

**Introduction:**

Surgeons have widely regarded sternotomy (ST) as the standard surgical method for thymectomy. Minimally invasive methods for thymectomy, including video-assisted and robot-assisted thoracoscopic surgery (RATS), have been explored. There are some studies have researched and compared the outcomes of patients after robotic and sternotomy procedure.

**Methods:**

We searched the databases of Pubmed, the Cochrane Library, Embase and selected the studies on the efficacy and safety of RATS or ST for thymectomy. Meta-analysis was performed for operation time, operation blood loss, postoperative drainage time, operative complications and hospitalization time.

**Results:**

A total of 16 cohort studies with 1,089 patients were included. Compared to ST, RATS is an appropriate alternative for thymectomy which reduced operation blood loss [standardized mean difference (SMD) = −1.82, 95% confidence interval (95% CI): (−2.64, −0.99), *p* = 0.000], postoperative drainage time [SMD = −2.47, 95% Cl: (−3.45, −1.48), *p* = 0.000], operative complications [odds ratio (OR) = 0.31, 95% Cl: (0.18, 0.51), *p* = 0.000] and hospitalization time [SMD = −1.62, 95% Cl: (−2.16, −1.07), *p* = 0.000].

**Conclusions:**

This meta-analysis based on cohort studies shows that RATS has more advantages over ST. Therefore, RATS is a more advanced and suitable surgical method for thymectomy.

## Introduction

Thymus is an important immune and endocrine organ in human body. Thymoma is an unusual thymic tumor. Its annual incidence rate in the population is about 0.15/100,000 (1). Surgical intervention is the only effective method for its treatment. In the past, median sternotomy was regarded as the first surgical approach for all types of thymomas, which ensured the safety of tumor resection. Sternotomy has been widely considered and applied to the standard surgical method of thymectomy. Because sternotomy is an invasive operation, the operation involves the incision of long bone, which may lead to complications such as intraoperative bleeding, postoperative pain and infection (2). Surgeons have explored many minimally invasive surgery approaches, including video-assisted and robot-assisted thoracoscopic surgery. In minimally invasive surgery, video-assisted thoracoscopic surgery (VATS) is the most popular and commonly used approach. Thoracoscopic surgery is considered to be the first choice for thymectomy because it can reduce intraoperative bleeding, postoperative pain and the incidence of postoperative complications (3–5). However, video-assisted thoracoscopy has some limitations. Thymectomy sometimes requires fine anatomy or complex surgery in the narrow upper mediastinum, which is technically challenging.

As an advanced minimally invasive surgery platform, robot-assisted surgery overcomes the limitations of traditional thoracoscopic surgery. The introduction and development of the da Vinci Robotic System has brought many obvious conveniences to surgeons, such as providing clear three-dimensional images, greater freedom of movement of surgical instruments in limited space, and reducing hand-related tremors. The da Vinci Robotic System also can help surgeons achieve more accurate anatomy, resulting in better clinical and tumor results, especially when thymectomy is performed in a narrow space (6). At present, it is not clear whether robot-assisted minimally invasive surgery can bring more benefits to doctors and patients. Many researchers have explored robotic treatment of thymic diseases, and some comparative studies on the surgical effects of robotic and sternotomy surgery have been published. The original purpose of this meta-analysis is to confirm the feasibility and safety advantages of robot-assisted thymectomy compared with sternotomy.

## Methods

### Search strategies

**Figure 1:**
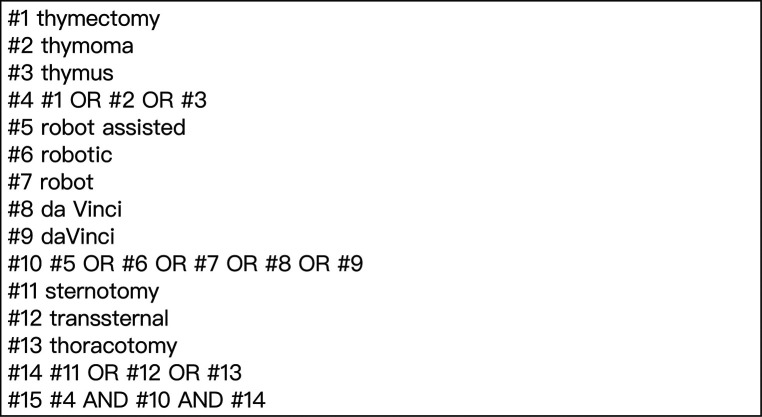
The search strategy.

We searched and identified relevant studies from the databases of Pubmed, the Cochrane Library, Embase (from the establishment time of database to August 2022). The search terms that related to thymectomy, sternotomy and robot assisted are as follows: “thymectomy”, “thymoma”, “thymus”, “sternotomy”, “transsternal”, “thoracotomy”, “robot assisted”, “robotic”, “robot”, “da Vinci” and “daVinci”. Figure 1 shows the search strategy. In addition, if we find other studies closely related to robot-assisted thoracoscopic thymectomy in other literatures, we will further search and evaluate them.

### Inclusion and exclusion criteria of studies

#### Inclusion criteria

(1) The English language journal study; (2) the study described robot-assisted surgery and sternotomy for thymectomy; (3) the study provided original data.

#### Exclusion criteria

(1) Article was not in English; (2) review, conference abstracts, or case report; (3) unable to extract data.

### Identification of literature

Three independent researchers reviewed titles or abstracts of the studies. The studies that meet the inclusion criteria were searched for full-text evaluation. The trials selected for detailed analysis were analyzed by three researchers, and disagreements were resolved by the fourth researcher.

### Collection of study indicators

The data that we collected included: (1) publication date and country of literature; (2) the number of subjects of each research; (3) the mean age of patients; (4) outcomes include: operation time, operation blood loss, postoperative drainage time, operative complications and hospitalization time.

### Quality assessment of included studies

We assessed the quality of all included studies from the perspectives of selection, comparability and exposure by the Newcastle–Ottawa Scale (NOS). The star system was used to score all studies, with a maximum of 9 stars. The specific evaluation criteria are that 8–9 stars represent high quality and 6–7 stars represent reasonable.

### Statistical methods and analysis

We used Stata/SE 17.0 software to estimate statistical significance. The odds ratio (OR) was used to assess binary variables and the standardized mean difference (SMD) was used to assess continuous variables. The identification of heterogeneity of studies was calculated by the *I*^2^ statistics. When the heterogeneity test result is significant (*I*^2^ > 50% or *p* < 0.05), a random-effect model was used to evaluate. Otherwise, a fixed-effect model was used. At the same time, publication bias was assessed by Egger's test and Begg's test.

## Results

### Study selection process

**Figure 2:**
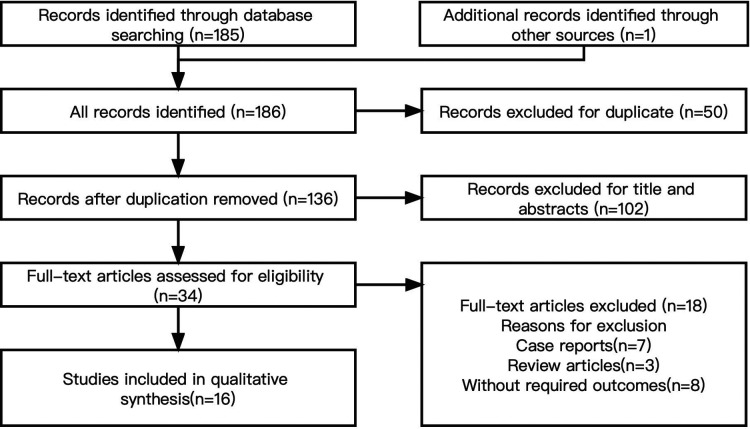
The study selection process.

We identified 186 studies, of which 16 (2, 6–20) were included in our analysis. All studies involved a total of 1,089 patients. Figure 2 shows the study selection process.

### Characteristics and quality of study

Table 1 shows the characteristics and quality of the studies.

**Table 1:** Included studies characteristics.

Study	Country	Study design	Number of patients		Mean age		Outcome	NOS
			RATS (M/F)	ST (M/F)	RATS	ST		
Cakar 2007 (7)	Austria	CS	9	10	–	–	①④	7
Balduyck 2011 (8)	Belgium	CS	14 (4/10)	22 (12/10)	49.0 (18.0–63.0)	56.0 (23–84)	①⑤	7
Weksler 2012 (9)	United States	CS	15 (7/8)	35 (18/17)	56.8 ± 16.3	50.7 ± 17.7	②④⑤	7
Renaud 2013 (10)	France	CS	6 (1/5)	15 (6/9)	40 (27–57)	27.9 (6–46)	①③⑤	7
Seong 2014 (11)	Korea	CS	34 (15/19)	34 (18/16)	53.7 ± 2.2	52.4 ± 1.8	①③④⑤	7
Ye 2014 (12)	China	CS	23 (11/12)	51 (31/20)	52.5 ± 7.4	50.1 ± 12.7	①②③④⑤	8
Kang 2016 (2)	Korea	CS	100 (48/52)	100 (51/49)	52.1 ± 13.6	52.3 ± 13.4	①②④	7
Wilshire 2016 (13)	United States	CS	23 (11/12)	17 (12/5)	58 (50–67)	59 (52–69)	①②③⑤	7
Kamel 2017 (14)	United States	CS	22 (8/14)	22 (9/13)	58 (50–67)	59 (51–72)	①②③④⑤	8
Kneuertz 2017 (15)	United States	CS	20 (5/15)	34 (14/20)	59 (47–65)	61 (47–73)	①②③④⑤	8
Qian 2017 (16)	China	CS	51 (21/30)	37 (15/22)	48.8 ± 13.3	46.8 ± 13.7	①②③④⑤	7
Casiraghi 2018 (6)	Italy	CS	24 (10/14)	24 (7/17)	61.6 ± 11.1	59.3 ± 11.5	①④⑤	7
Marulli 2018 (17)	Italy	CS	41 (18/23)	41 (19/22)	58.24 ± 10.97	57.66 ± 10.30	①③④⑤	7
Ancin 2019 (18)	Turkey	CS	12	16	31.5 (28.25–40.00)	41.50 (37.35–45.75)	①③⑤	7
Imielski 2020 (19)	United States	CS	54 (29/25)	69 (38/31)	44.9 ± 15.8	53.2 ± 16.8	①④⑤	7
Luzzi 2021 (20)	Italy	CS	57 (22/35)	57 (27/30)	50.8 (18–81)	54 (11–82)	①⑤	7

M, male; F, female; CS, cohort study; ① operation time, ② operation blood loss, ③ postoperative drainage time, ④ operative complications, ⑤ hospitalization time.

### Analysis results

#### Operation time

**Figure 3:**
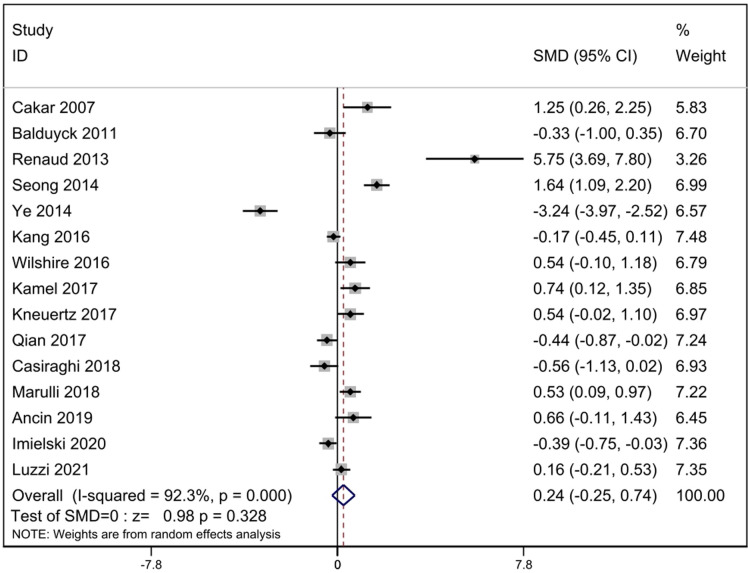
Comparison of operation time between RATS and ST. SMD, standardized mean difference; CI, confidence interval; RATS, robot-assisted thoracoscopic surgery; ST, sternotomy.

Fifteen studies reported operation time. According to the heterogeneity test results, it can be concluded that statistical heterogeneity was significant between the fifteen studies (*p* = 0.000, *I*^2^ = 92.3%), we used random-effect model for calculation. The data revealed that significant difference did not exist between the RATS and the ST [SMD = 0.24, 95% CI: (−0.25, 0.74), *p* = 0.328] (Figure 3).

#### Operation blood loss

**Figure 4:**
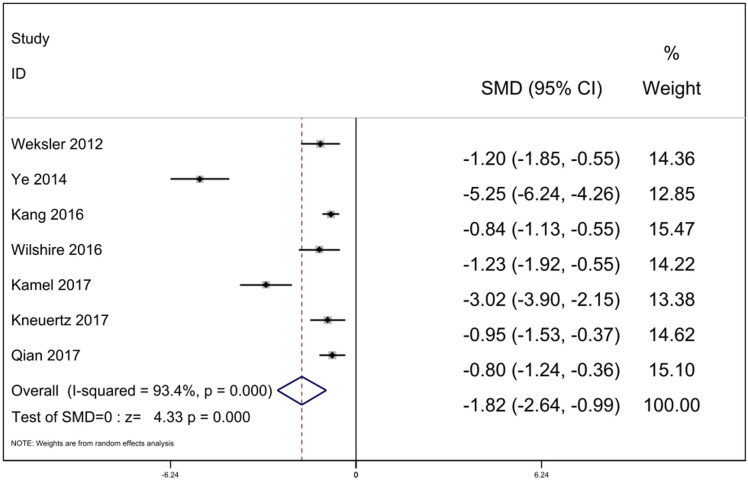
Comparison of operation blood loss between RATS and ST. SMD, standardized mean difference; CI, confidence interval; RATS, robot-assisted thoracoscopic surgery; ST, sternotomy.

Operation blood loss was compared in seven studies. According to the heterogeneity test, it can be concluded that statistical heterogeneity was significant between the seven studies (*p* = 0.000, *I*^2^ = 93.4%), we calculated by random-effect model. The result revealed that operation blood loss was less in the RATS group [SMD = −1.82, 95% Cl: (−2.64, −0.99), *p* = 0.000] (Figure 4).

#### Postoperative drainage time

**Figure 5:**
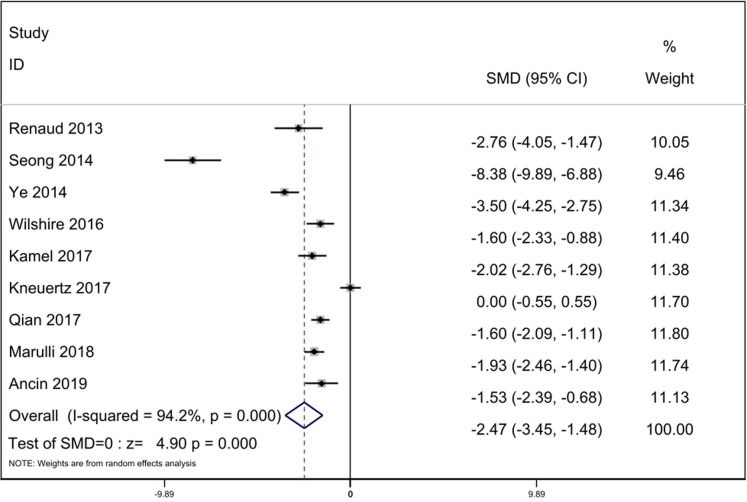
Comparison of postoperative drainage time between RATS and ST. SMD, standardized mean difference; CI, confidence interval; RATS, robot-assisted thoracoscopic surgery; ST, sternotomy.

Nine studies reported complete data of postoperative drainage time. The statistical heterogeneity was significant in the nine studies. We uesd the random-effect model for calculation (*p* = 0.000, *I*^2^ = 94.2%). The result indicated that postoperative drainage time were less in the RATS group [SMD = −2.47, 95% Cl: (−3.45, −1.48), *p* = 0.000] (Figure 5).

#### Operative complications

**Figure 6:**
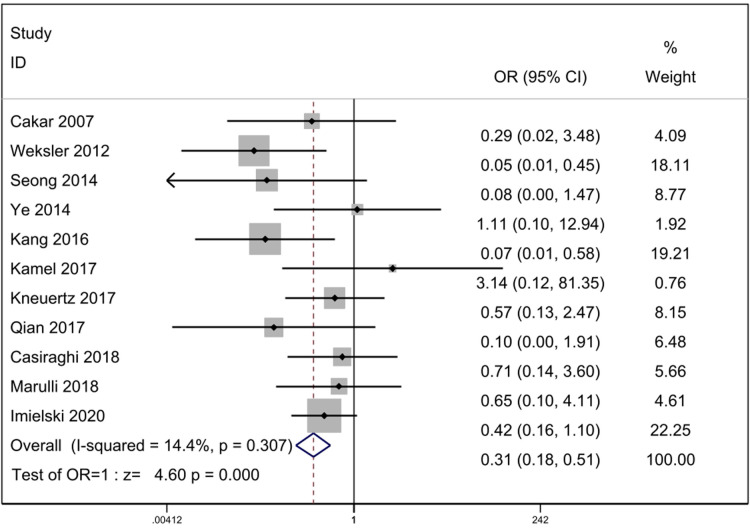
Comparison of operative complications between RATS and ST. OR, odds ratio; CI, confidence interval; RATS, robot-assisted thoracoscopic surgery; ST, sternotomy.

According to the heterogeneity test results, it can be concluded that statistical heterogeneity was not significant between the eleven studies (*p* = 0.307, *I*^2^ = 14.4%), we adopted fixed-effect model for calculation. The data revealed that operative complications was less in RATS group. [OR = 0.31, 95% Cl: (0.18, 0.51), *p* = 0.000] (Figure 6).

#### Hospitalization time

**Figure 7:**
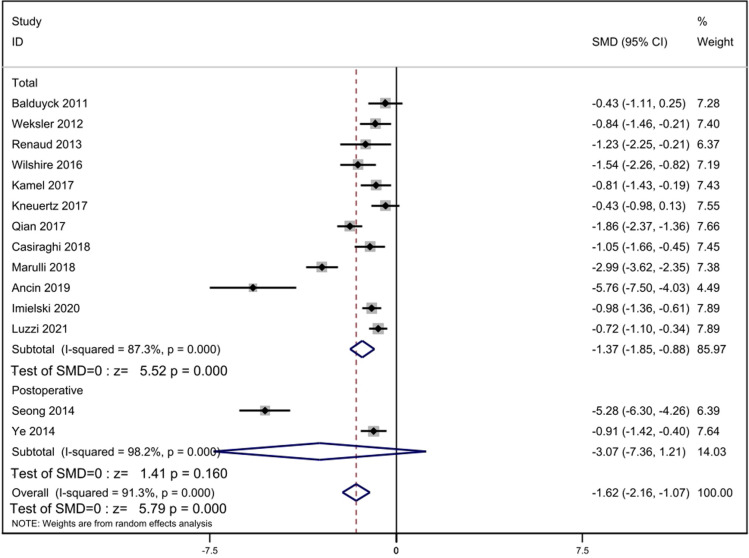
Comparison of hospitalization time between RATS and ST. SMD, standardized mean difference; CI, confidence interval; RATS, robot-assisted thoracoscopic surgery; ST, sternotomy.

Fourteen studies with complete data compared hospitalization time. Statistical heterogeneity was significant (*p* = 0.000, *I*^2^ = 91.3%). We used the random-effect model for calculation. The result indicated that hospitalization time was less in the RATS group [SMD = −1.62, 95% Cl: (−2.16, −1.07), *p* = 0.000]. There were twelve studies reported total hospitalization time and two studies reported postoperative hospitalization time. We performed subgroup analysis and found that total hospitalization time was less in the RATS group [SMD = −1.37, 95% Cl: (−1.85, −0.88), *p* = 0.000], but postoperative hospitalization time was similar in two groups [SMD = −3.07, 95% Cl: (−7.36, 1.21), *p* = 0.160] (Figure 7).

### Assessment of publication bias

**Figure 8:**
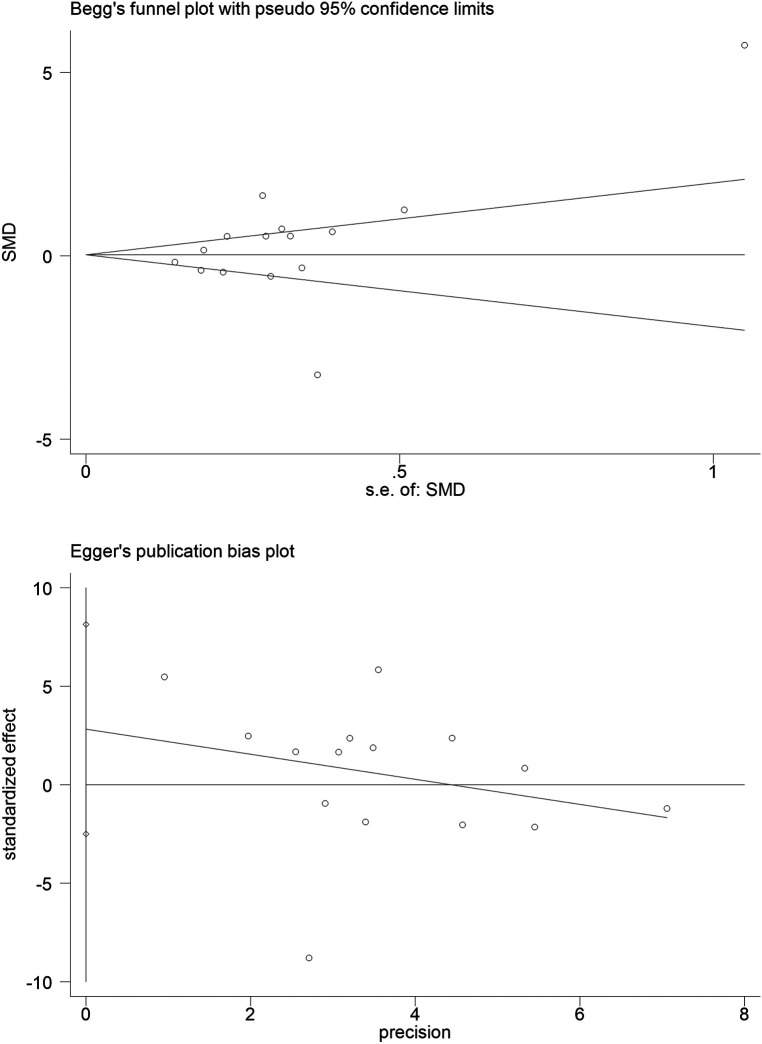
Assessment of publication bias. Begg's test and Egger's test did not imply a publication bias.

The Begg's test (*z* = 1.29, Pr > |*z*| = 0.198) and the Egger's test (*t* = 1.15, *p* > |*t*| = 0.273) revealed that publication bias did not exist in these included studies, and the results of this meta analysis are stable (Figure 8).

## Discussion

The best surgical method of thymectomy has always been a controversial issue. So far, sternotomy has been regarded as the first choice for thymectomy, especially for thymoma. Surgeons can expose the entire mediastinum in this way to get the best surgical field of vision. In the era of rapid development of artificial intelligence, the introduction of robotic surgery system has brought a valuable choice to doctors. The daVinci robot has all the advantages of minimally invasive surgery. It provides a clearer, three-dimensional 3D field of vision than video-assisted thoracoscopy, reduces the impact of surgeons' hand tremors, and makes the movement of instruments more accurate (21, 23). Many original studies have explored robot-assisted thymectomy for the treatment of thymic diseases, some scholars have studied the surgical results of patients after robotic surgery and sternotomy. Therefore, a meta-analysis was performed to confirm the advantages of robot-assisted thoracoscopic surgery for thymectomy.

From our meta-analysis, it can be concluded that compared with ST, RATS thymectomy has obvious advantages, including less operative blood loss, less drainage time, less postoperative complications and less hospitalization time. The comparison of operation time was not significant.

Our meta-analysis revealed that the significant difference did not exist in operation time between RATS and ST. For surgeons, robotic-assisted surgery has a learning curve, so the operation time may be affected by the surgeons' technology. With the improvement of the surgeons' surgical technology, the operation time will be reduced (11, 24). The comparison results of operation time had significant heterogeneity (*I*^2^ = 92.3%). Based on the sensitivity analysis, we conducted that the studies of Ye (12), Renaud (10) and Seong (11) caused the heterogeneity. After reviewing the full texts carefully, there was no significant difference between these three studies and the other fifteen studies. We eliminated the three studies, the heterogeneity decreased slightly (*I*^2 ^= 73.6%). We speculate that although the general surgical procedures are roughly the same, there are great differences in surgical skills among different institutions, resulting in temporal heterogeneity. Of course, it is also possible that the definition of operation time is different in different studies. some studies define the startup of the robot system as the start time, some count the operation time according to the anesthesia time, and some choose the skin-to-skin time. These may help us to understand the heterogeneity of this result.

From the results of our meta-analysis, operation blood loss of RATS group was less compared with ST group (*p* = 0.000). We speculate that during the operation, the robot can provide surgeons with clearer three-dimensional images, and its flexible operating arm can avoid hand tremors, help doctors more effectively separate the complex anatomical structures of the chest and accurately expose the thymus, and help surgeons perform accurate operations (25). We observed significant heterogeneity of intraoperative blood loss (*I*^2^ = 93.4%), and our sensitivity analysis showed that the study of Ye (12) was most likely to lead to heterogeneity. After excluding the study, the heterogeneity decreased (*I*^2^ = 78.5%).

With regard to the postoperative drainage time, the result of heterogeneity test is significant (*I*^2^ = 94.2%). The sensitivity analysis was performed and suggested that the heterogeneity was caused by three studies by Seong (11), Ye (12) and Kneuertz (15). We eliminated the studies, the heterogeneity disappeared (*I*^2^ = 0%). After reviewing the full texts carefully, We found no significant difference between these three studies and the other six studies. Therefore, we speculated that there are differences in the indicators of removing drainage tube in different institutions, which may explain the heterogeneity of postoperative drainage time. Our analysis suggested that the postoperative drainage time of RATS was less compared with ST (*p* = 0.000).

Operative complications are related to the recovery of patients. Our meta analysis indicated that for thymectomy, robotic surgery had a lower incidence of operative complications than sternotomy. This result is due to the fact that robotic surgery provides a clear field of vision and precise manipulation, which can reduce tissue damage and reduce complications including postoperative pain and infection.

As for hospitalization time, the analysis suggested that hospitalization time of patients in RATS group was shorter. The result is observably attributed to the minimally invasive characteristics of robot-assisted surgery, which can avoid tissue injury, reduce intraoperative blood loss, shorten the time of pleural drainage days and accelerate the postoperative recovery of patients. The heterogeneity of hospitalization time was significant (*I*^2^ = 91.3%). Through the sensitivity analysis, we can conclude that the heterogeneity was mainly caused by the studies of Seong (11), Marulli (17) and Ancin (18). Heterogeneity decreased after the elimination of the two studies (*I*^2^ = 56.2%).

Shen et al. (26) and Wu et al. (27) compared the effects of RATS and VATS thymectomy by meta-analysis. They all came to a similar conclusion: RATS has more advantages over VATS, including reducing operation blood loss, postoperative drainage time, postoperative drainage volume, hospitalization time, and postoperative complications. It can be concluded that compared with ST and VATS, RATS is a more suitable surgical method for thymectomy.

The operation field of traditional video-assisted thoracoscopy is two-dimensional, and the field is not clear enough. The robot surgery operating system adds a new dimension, and its camera system can achieve a 10-fold magnification of the surgical field of vision, which helps surgeons to observe complex and small structures in more detail. The flexibility of the robot system is significantly higher than that of traditional surgical instruments, and its surgical arm can flexibly perform complex three-dimensional operations, overcoming some technical and methodological limitations. During thymectomy, the clear, flexible and stable characteristics of the robot system can ensure the structural integrity of blood vessels and nerves which are often damaged (28, 29).

What we need to admit is that our meta-analysis has some limitations. First of all, the studies we searched and included are cohort studies. There is no randomized controlled trial concerning the clinical difference between RATS and ST in databases at present. We will focus on randomized controlled trials in the future so that we can update this meta-analysis.

## Conclusion

According to this meta-analysis of cohort studies, it can be concluded that RATS has more advantages over ST, including reducing operation blood loss, postoperative drainage time, incidence of operative complications and hospitalization time. Therefore, robot-assisted thoracoscopic surgery is a more appropriate surgical option for thymectomy.

## Data Availability

The original contributions presented in the study are included in the article/Supplementary Material, further inquiries can be directed to the corresponding author/s.
